# Aluminum Concentrations in Male and Female Football Players during the Season

**DOI:** 10.3390/toxics11110920

**Published:** 2023-11-10

**Authors:** María C. Robles-Gil, Víctor Toro-Román, Marcos Maynar-Mariño, Jesús Siquier-Coll, Ignacio Bartolomé, Francisco J. Grijota

**Affiliations:** 1 Faculty of Sport Sciences, University of Extremadura, 10003 Cáceres, Spain; mcroblesgil@unex.es (M.C.R.-G.); mmaynar@unex.es (M.M.-M.);; 2 Department of Communication and Education, University of Loyola Andalucía, 41704 Sevilla, Spain; jsiquier@alumnos.unex.es; 3 Faculty of Health Sciences, Isabel I University, 09003 Burgos, Spain; ignbs.1991@gmail.com; 4 Faculty of Education, Pontifical University of Salamanca, 37007 Salamanca, Spain

**Keywords:** urine, plasma, erythrocyte, athletes, sex, training

## Abstract

Aluminum (Al) is one of the most abundant trace mineral elements in the earth’s crust. Al is considered a potent neurotoxicant. Physical exercise could cause modifications in some trace mineral elements. On the other hand, there could be sex differences in the exposure and deposits of toxic mineral elements. The aim of the present study was to compare sex and seasonal differences in extracellular and intracellular Al concentrations in football players. The study involved 22 male and 24 female football players from the fifth and second national category, respectively. Three assessments were carried out during the season (beginning, middle and end). Al concentrations in plasma, urine, erythrocytes and platelets were determined. Male football players ingested more Al (*p* < 0.05). Higher plasma Al concentrations were reported in male football players (*p* < 0.01). On the other hand, in both groups, increases and decreases in Al in the plasma and urine were observed in the second and third assessment, respectively (*p* < 0.01). There were sex differences in platelet Al concentrations (*p* < 0.05). Plasma and platelet Al concentrations may be different between the sexes. Al concentrations may change over the course of a season in football players.

## 1. Introduction

Trace mineral elements (TMEs) are inherently found in the environment and people are exposed to these elements through various media such as air, water and food.

Environmental pollution is associated with an increased likelihood of developing cardiovascular disease [1], respiratory disease [2], increased oxidative stress [3], systemic inflammation [1] and cancer [4]. These conditions have a negative impact, especially on athletes as they can lead to a decrease in their performance [5].

Aluminum (Al) is one of the most abundant TMEs in the earth’s crust [6]. Although Al has not been shown to play an important role in humans, it has effects on growth, reproduction and milk production in some animal studies [7]. Specifically, Al is a potent neurotoxicant. The neurotoxicity of Al has been demonstrated in humans, animal models and in tissue and cell cultures [8]. There are countless ways in which Al can exert toxicity. The ^3+^Al ion is highly biologically reactive. However, the concentration in any compartment must reach a threshold [9].

Humans are experiencing increasing Al exposure with concomitant effects on their body burden. Systemic Al is the sum of all Al associated with the inside and outside of the body at any time and is significantly influenced by excretion. It is currently assumed that urine is the main route of excretion [10].

One of the measures that helps to minimize toxic mineral concentrations is regular physical exercise [11]. Physical exercise is known to cause changes in the concentrations of essential [12,13,14] and toxic TMEs [11,15]. Regarding Al, the literature concerning the influence of physical training is scarce. An investigation by Otag et al. (2014) reported decreases in serum Al during a maximal aerobic test [16]. The decrease continued even after exercise, suggesting that it may have positive clinical and performance effects.

It is important to focus research on possible interactions between sex and the effects of environmental exposure to toxic minerals on health. Because of this, sex differences in toxic mineral exposure and deposition have been studied by authors such as Vahter et al. (2007) [17] and Keitt et al. (2004) [18]. The health effects of certain toxic TMEs manifest themselves differently in men and women due to differences in kinetics and mode of action [17]. However, information is scarce regarding sex differences in Al concentrations. It is speculated that Fe status, as well as other toxic minerals [19], might influence Al uptake, influencing the evolution of neurodegeneration [20].

Due to the lack of information on the influence of physical exercise on Al concentrations in men and women, the present study aimed to analyze the differences between sexes in Al concentrations in different biological matrices (plasma, urine, erythrocytes and platelets) and to analyze their evolution throughout a sports season.

## 2. Materials and Methods

### 2.1. Study Design

**Figure 1:**
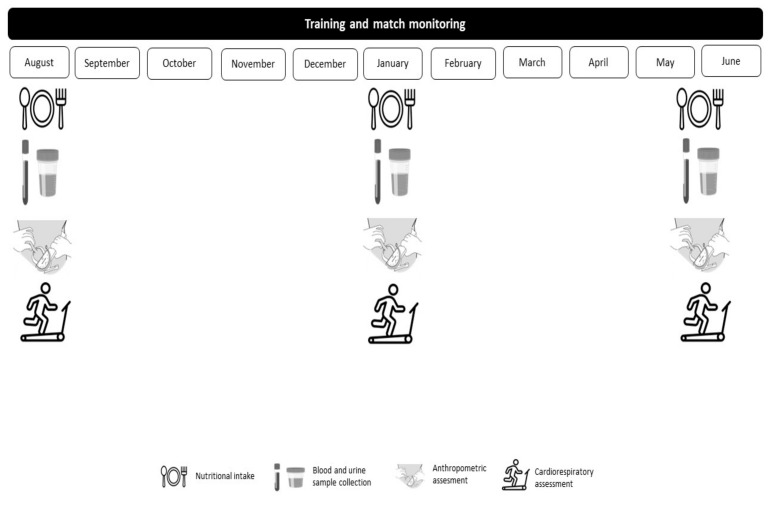
Study design.

The present study was accepted and approved by the bioethics committee of the University of Extremadura (135/2020). The protocol was similar to that reported by Toro-Román et al. [21,22]. A longitudinal study of approximately 11 months duration was carried out. Three assessments were conducted throughout the sport season (August–June): assessment 1 (start of season; August); assessment 2 (middle of season; January); and assessment 3 (end of season; May–June) (Figure 1).

At the beginning of the study, different assessments were carried out to characterize the sample. On the other hand, nutritional assessment, blood extractions and urine collections were performed the same week of each month, in the morning and in the same order for all subjects.

### 2.2. Participants

All subjects agreed to participate in the present study after being informed about the purpose of the study and signing a consent form.

The sample of the present investigation consisted of a total of 46 football players divided into male football players (n = 22) and female football players (n = 24). All participants trained and competed in the same city (Table 1). Data concerning training characteristics were provided by the team staff.

Subjects had to meet the following criteria: (i) residence in the same city before and during the study; (ii) absence of pathologies; (iii) no medication or supplementation during the study period and the previous month; (iv) no smoking or drug use; (v) more than 5 years of experience playing soccer; (vi) no change in nutritional and physical activity habits during the study; and (vii) no more than 30 days without training with the team. In addition, the women had to meet the following criteria: (viii) have regular menstrual cycles for at least six months before the start of the study and during the study; (ix) not suffer from problems related to their menstrual cycle; and (x) not use contraceptive methods.

A control of the matches played and the training sessions was carried out in order to know some data on the external load of both teams throughout the sports season (Table 1). The internal load could not be added to this document since each coaching staff used different methodologies that could not be compared.

### 2.3. Nutritional, Anthropometric and Cardiorespiratory Assessment

This section is similar to that reported by Toro-Román et al. [19,21].

To ascertain Al intake, participants completed a nutritional questionnaire. The nutritional composition of each food was assessed by indicating the amount and frequency of food intake during the 3 days prior to the assessments [23].

Anthropometric assessments were carried out in accordance with the guidelines of Porta et al. [24]. The following assessments were made: height (Seca 220. Hamburg, Germany), body mass (Seca 769. Hamburg, Germany) and skinfolds (abdominal, suprascapular, subscapular, tricipital, thigh and calf) (Holtain© 610ND skinfold plicometer).

A maximal incremental test was performed on a treadmill (Ergofit Trac Alpin 4000, Pirmasens, Germany), equipped with a gas analyzer (Geratherm Res-piratory GMBH, Ergostik, Ref 40.400, Corp, Bad Kissingen, Germany) and a Polar heart rate monitor (Polar^®^ H10, Kempele, Finland). Participants ran for 1 min periods until exhaustion. The test started at 7 km/h and increased by 1 km/h every minute with a 1% gradient. Before the test, a 15 min warm-up was performed (Table 2).

### 2.4. Blood and Urine Sample Collection and Determination of Hematological Parameters

Blood draws began at 8:00 am on an empty stomach. Subjects came with the first urine of the day. Approximately 9 mL of urine was frozen at −80 °C until analysis.

A total of 12 mL of blood was drawn with a 20 mL plastic syringe. Of the total, 2 mL was collected to determine hematological parameters (Coulter Electronics LTD, Model CPA; Northwell Drive, Luton, UK). The remaining 10 mL were used to determine Al concentrations. Two 4 mL samples were collected with sodium citrate. For plasma, one Vacutainer^®^ tube with sodium citrate was taken and centrifuged at 1800 rpm for 8 min. The platelet-rich plasma was collected in dry BD Vacutainer^®^ tubes and centrifuged for 10 min at 3000 rpm. The plasma was divided into 1.5 mL Eppendorf tubes and allowed to stand at −80 °C. To the adherent platelets, 1 mL of pure water (Mili-Q) was added and vortexed (Cole-Parmer™, Stuart™, Vernon Hills, IL, USA). The mixture was transferred to an Eppendorf tube and stored at −80 °C. Erythrocytes were removed from the remaining blood and washed three times with 0.9% sodium chloride. They were then collected into Eppendorf tubes and stored at −80 °C.

### 2.5. Determination of Al

Al determination was performed by inductively coupled plasma mass spectrometry (ICP-MS) (7900; Agilent Tech., Santa Clara, CA, USA). The linearity of the calibration curves for indium (In) was greater than 0.985.

For plasma and urine samples, the reagents used were 69% nitric acid and ultrapure water obtained from a Milli-Q system (Millipore^®^, Burlington, MA, USA). A rhodium dilution of 400 µg/L was used as the internal standard. From the 0.20 mL of samples, a volume of 5 mL was made up with a 1% nitric acid solution prepared from a 69% commercial solution (TraceSELECT™, Fluka™, Madrid, Spain). The kit was calibrated with several certified prepared standards.

For erythrocyte and platelet samples, the reagents used were 69% nitric acid, hydrogen peroxide (TraceSELECT™, Fluka™, Madrid, Spain) and ultrapure water obtained from a Milli-Q system. A 400 µg/L solution of yttrium and rhodium was used as the internal standard.

Samples were weighed on a precision balance and transferred to glass microwave digestion tubes, and 3.5 mL of a 3:1 mixture of 69% nitric acid (TraceSELECT™, Fluka™, Madrid, Spain) and hydrogen peroxide (TraceSELECT™, Fluka™) was added. Samples were digested in a Milestone Ultrawave microwave and, once digested, were diluted to 25 mL with MilliQ water (Table 3).

### 2.6. Statistical Analysis

The data were processed with the IBM SPSS 25.0 statistical program (IBM Corp., Armonk, NY, USA). The normality of the variables was analyzed using the Shapiro–Wilk test. A two-way ANOVA (sex effect and time effect) was used to determine the differences. Bonferroni post hoc test was applied. Differences of *p* < 0.05 were considered statistically significant.

## 3. Results

The results obtained in the present study are shown below. Table 4 shows the data for the total energy and Al intake throughout the study. There were significant differences between the sexes in the total energy and Al intake (*p* < 0.05).

Table 5 shows the data concerning erythrocytes and platelets. There were sex differences in the number of red blood cells. There were also significant differences across the season. Specifically, there were differences between assessments 1 and 2 and 1 and 3 (*p* < 0.01).

Table 6 shows plasma and urinary Al concentrations throughout the season in both sexes. There were sex differences in plasma concentrations. On the other hand, there were seasonal differences in plasma and urinary concentrations. In plasma, differences were found between measurements 1 and 2 (*p* < 0.01) and 2 and 3 (*p* < 0.01). For urinary concentrations, there were differences between assessments 1 and 2 (*p* < 0.01) and 2 and 3 (*p* < 0.01).

According to Table 7, there were differences between the sexes in the platelet concentrations of Al with *p* < 0.05. Additionally, there were significant differences observed in the relative erythrocyte concentrations, absolute platelet concentrations, and relative platelet concentrations throughout the evaluations with *p* < 0.05. There were also differences observed in erythrocyte concentrations between evaluations 2 and 3 with *p* < 0.05 and in platelet concentrations between evaluations 1 and 2 and 1 and 3 with *p* < 0.01.

## 4. Discussion

The objectives of the research were (i) to analyze sex differences in Al concentrations in different biological matrices (plasma, urine, erythrocytes and platelets) and (ii) to analyze changes in Al concentrations during a season in soccer players. The novelty of the present study is in the longitudinal and between-sex comparison of Al concentrations in different biological matrices. The results show differences between sexes in Al concentrations, as in the study carried out by Vahter et al. (2007) [17] on different toxic metals such as cadmium or lead [17]. The present study reinforces the hypothesis that there are gender differences in the kinetics and disposition of toxic minerals [19]. Only one study has investigated the influence of physical exercise on Al concentrations in athletes (elite wrestlers) and specifically in a single compartment such as serum [16].

The Al concentrations found in our study, both in men and women, are within similar ranges to those estimated in a recent population-based study in urine, which was conducted in 102 sedentary individuals [25]. However, Merian et al. (2004) [26] established that healthy humans contain Al concentrations at the level of 3–9 μg/L in serum and urine, respectively, which are values well below those found in our study. Despite this, the same author states that Al toxicity is of concern when the serum level is higher than 200 μg/L. In our study, the plasma Al concentrations in men and women were considerably lower than those established in the above-mentioned study.

There is some debate about the effect of Al in the human body, but there are no clear conclusions. Air, water and different types of food, especially preserved food or food with additives, are sources of Al [27]. It is well documented that Al is a neurotoxic metal [28] and there are important studies such as Islam et al. (1999) [29] who described the neurodegenerative disorder known as motor neuron disease among 17–30 year olds due to prolonged Al exposure [29]. Also, kidney patients undergoing dialysis treatment are exposed to high concentrations of Al, which lead to neurotoxicity and bone disease [30]. However, a part of the scientific community maintains that Al does not manifest direct toxic effects and does not induce the formation of free radicals (ROS) [31], as considered by other authors [32]. Al intake must be considered. In the present study, both groups ingested lower amounts than those established as toxic (2000 μg/kg) [26]. In the present study, men ingested higher amounts of Al compared to women. In general, adults consume 1–10 mg Al per day from natural sources [33]. On the other hand, cooking in Al containers often results in statistically significant increases. We have found no information to justify gender differences in Al intake.

Considering changes over (longitudinal) assessments in the present study, plasma and urinary Al concentrations were reported to increase at the second assessment (*p* < 0.01) and to decrease at the end of the study above baseline values (*p* < 0.01). In non-professional American football players, urinary Al concentrations were determined before a training period (8 sessions) and after the training period. After the training period, urinary concentrations increased in our study between assessments 1 and 2, and these increases were significant (*p* < 0.01) [34]. In another study carried out on elite wrestlers, serum Al concentrations were measured before training, immediately after training and one hour after training. The serum Al level decreased during exercise and this decrease continued even after exercise, suggesting to the authors that these changes could have positive effects in terms of clinical and exercise performance [16].

Increases in plasma and urinary Al could be due to different reasons. On the one hand, it may be related to the progressive increase in Al intake. On the other hand, several mechanisms, including increased renal excretion, are related to physical activity and/or increased body absorption by inhalation due to increased ventilation rate, in parallel to an increased excretion [34]. The increased excretion of Al could be the body’s response to enhance the elimination of this toxic metal, preventing its accumulation. In relation to the above, it has been suggested that men, through perspiration, excrete Al more efficiently compared to women and that regular physical exercise could be a way to increase Al excretion [35]. On the other hand, regarding sex differences, several studies have reported that Al exhibits the same effect on transferrin receptors (TfRs) and ferritin as Fe in the deficiency state. Thus, Al increases the number of TfRs, leading to increased Fe uptake and also decreases ferritin, which could result in higher levels of free Fe. These effects of Al on Fe homeostasis could explain the increased oxidative stress and inflammation both in vitro and in vivo following Al exposure [36]. Young women showing low levels of Fe storage would be more able to store Al but would be protected from Al-Fe interactions and thus oxidative stress. Conversely, postmenopausal women would have higher Fe stores, but Al stores would remain elevated [20].

Regarding intracellular Al concentrations, there were sex differences in platelet Al concentrations. On the other hand, there was an increase in Al in erythrocytes in men in the second evaluation compared to the first, and a decrease in the third assessment below even the initial values. In women, Al concentrations decreased in the second assessment, with a much more pronounced decrease in the third evaluation. Finally, in platelets, in both men and women, there was a significant decrease in Al concentrations between the first and second assessments. However, in the third evaluation, there was an increase in concentrations in both sexes compared to the second evaluation. Unfortunately, we did not find information on Al concentrations in erythrocytes and platelets. Al causes numerous changes in the peripheral blood and hematological system. It also causes normo- or microcytic anemia, as it alters the maturation of cells of the erythroblastic series and heme biosynthesis [37]. In rats, high doses of Al interfered with different stages of RBC and mature RBC synthesis [38]. Therefore, it is important that Al concentrations are reduced in erythrocyte cells. Perhaps, as observed in the present investigation, regular training over time could be an interesting tool to reduce Al concentrations.

The present study has several limitations: (i) the small sample size; (ii) the absence of previous studies with which to compare the results; (iii) the measurement error was not analyzed; and (iv) Al concentrations in air and water in the area were not assessed. However, all participants lived in the same city.

## 5. Conclusions

Plasma, urinary and platelet concentrations of Al are different according to sex. Absolute platelet Al concentrations, as well as urinary and plasma concentrations, are higher in male soccer players.

Extracellular (plasma and urine) and intracellular (erythrocytes) concentrations of Al change throughout the sports season in football players.

It is important to know the intake of TME throughout a sport season to keep toxic elements below toxicity levels and to avoid possible losses in sport performance.

Sex differences could be related to Al intake. On the other hand, differences throughout the season could be related to increased inhalation due to a higher ventilation rate, parallel to a higher excretion.

## Figures and Tables

**Table 1:** Training and match control.

Parameters	Male (n = 22)	Female (n = 24)
Total Training (weeks)	36	39
Total Training (nº)	128.27 ± 18.59	133.54 ± 25.86
Total Training (min)	11,814.23 ± 1673.40	10,578.46 ± 3227.80
Absence from training (days)	12.07 ± 9.34	14.14 ± 10.79

**Table 2:** Participant´s characteristics.

Parameters	Male (n = 22)	Female (n = 24)
Age (years)	20.61 ± 2.66	23.37 ± 3.95
Experiences (years)	14.73 ± 3.31	14.51 ± 4.94
Training (sessions)	128.27 ± 18.59	133.54 ± 25.85
Height (m)	1.76 ± 0.06	1.65 ± 0.06
Weight (kg)	71.50 ± 5.93	59.58 ± 7.17
Σ6 Skinfold (mm)	60.34 ± 12.35	94.62 ± 18.54
VO_2max_ (L/min)	2.10 ± 0.20	1.40 ± 0.25
VE (L/min)	134.73 ± 13.75	81.40 ± 15.89
HR_max_ (bpm)	187.78 ± 6.52	183.33 ± 7.34

VO_2max_: maximal oxygen uptake; VE: expired volume; HR: heart rate.

**Table 3:** Erythrocyte sample digestion program.

Time (min)	Temperature (°C)	Power (W)	Pressure (bar)
5	100	1200	70
7	130	1300	100
8	170	1300	120
10	200	1500	120
15	200	1400	120

**Table 4:** Total energy and Al intake during the sports season.

Parameters	Time	Male (n = 22)	Female (n = 24)	Sex Effect	Time Effect	Sex × Time
Energy (kcal)	1	1796 ± 420	1578 ± 316	0.038	0.497	0.317
	2	1932 ± 312	1681 ± 427			
	3	1882 ± 358	1697 ± 386			
Al (µg/kg)	1	1040 ± 894	696 ± 512	0.031	0.136	0.261
	2	1118 ± 807	725 ± 451			
	3	1195 ± 808	751 ± 322			

Al: aluminum; mean ± standard deviation.

**Table 5:** Hematological parameters during the season.

Parameters	Time	Male (n = 22)	Female (n = 24)	Sex Effect	Time Effect	Sex × Time
Erythrocytes (10^6^)	1	4.92 ± 0.36	4.37 ± 0.22	<0.001	0.031	0.063
	2	4.83 ± 0.32 **	4.19 ± 0.27 **			
	3	4.99 ± 0.29 ++	4.35 ± 0.27 ++			
Platelets (10^3^)	1	204.50 ± 57.65	196.00 ± 38.01	0.274	0.542	0.222
	2	196.60 ± 39.79	219.08 ± 34.19			
	3	195.13 ± 37.82	204.39 ± 31.52			

**: *p* < 0.01 differences 1 vs. 2; ++: *p* < 0.01 differences 1 vs. 3; mean ± standard deviation.

**Table 6:** Al concentrations in plasma and urine.

Parameters	Time	Male (n =22)	Female (n = 24)	Sex Effect	Time Effect	Sex × Time
Plasma (µg/L)	1	2.92 ± 0.79	0.82 ± 0.21	<0.001	<0.001	0.141
	2	3.83 ± 0.75 **	2.90 ± 0.77 **			
	3	1.92 ± 0.23 ##	1.35 ± 0.26 ##			
Urine (µg/L)	1	22.08 ± 10.19	18.36 ± 11.57	0.295	<0.001	0.528
	2	34.71 ± 5.30 **	31.08 ± 16.40 **			
	3	27.84 ± 10.48 ##	26.46 ± 8.02 ##			

**: *p* < 0.01 differences 1 vs. 2; ##: *p* < 0.01 differences 2 vs. 3; mean ± standard deviation.

**Table 7:** Al concentrations in erythrocytes and platelets (absolute and relative values to cell number).

Parameters	Time	Male (n = 22)	Female (n = 24)	Sex Effect	Time Effect	Sex × Time
Erythrocytes (µg/L)	1	145.30 ± 116.31	149.87 ± 93.81	0.680	0.060	0.494
	2	191.80 ± 220.57	144.72 ± 158.24			
	3	96.24 ± 86.95 #	110.29 ± 81.62 #			
Erythrocytes (pg/cell^6^)	1	34.02 ± 25.99	35.40 ± 22.00	0.935	0.043	0.573
	2	39.40 ± 43.86	33.02 ± 37.45			
	3	18.93 ± 13.30 #	25.17 ± 18.51 #			
Platelets (µg/L)	1	109.61 ± 81.53	183.19 ± 99.57	0.007	<0.001	0.167
	2	84.86 ± 20.21 **	86.55 ± 21.39 **			
	3	101.19 ± 10.63 ++	103.87 ± 20.96 ++			
Platelets (pg/cell^3^)	1	0.850 ± 0.750	0.990 ± 0.638	0.716	<0.001	0.540
	2	0.447 ± 0.136 **	0.402 ± 0.121 **			
	3	0.538 ± 0.116 ++	0.522 ± 0.116 ++			

**: *p* < 0.01 differences 1 vs. 2; ++: *p* < 0.01 differences 1 vs. 3; #: *p* < 0.05 differences 2 vs. 3; mean ± standard deviation.

## Data Availability

Data are contained within the article.
